# Update on fluid biomarkers for concussion

**DOI:** 10.2217/cnc-2015-0002

**Published:** 2016-02-18

**Authors:** Henrik Zetterberg, Huw R Morris, John Hardy, Kaj Blennow

**Affiliations:** 1 Clinical Neurochemistry Laboratory, Institute of Neuroscience & Physiology, Sahlgrenska Academy, University of Gothenburg, Mölndal, Sweden; 2 Department of Molecular Neuroscience, UCL Institute of Neurology, Queen Square, London, UK; 3 Department of Clinical Neuroscience, UCL Institute of Neurology, Queen Square, London, UK

**Keywords:** biomarker, blood, cerebrospinal fluid, concussion, plasma

## Abstract

Concussions are difficult to diagnose and symptoms may not appear immediately. As an accurate initial diagnosis has profound implications for the clinical management, there is an unmet need for better diagnostic tools. Fluid biomarkers for CNS injury may represent such tools. These markers are often proteins, peptides or other molecules with selective or high expression in the brain, which can be measured in the cerebrospinal fluid or blood as they leak out or get secreted into the biofluid in response to the injury. Here, we review the literature on fluid markers of neuronal, axonal and astroglial injury and response mechanisms to diagnose CNS injury upon head impact and to determine when the injurious process has resolved.

Severe traumatic brain injury (sTBI) is easily diagnosed by clinical examination and standard neuroimaging techniques. However, mild TBI (mTBI) or concussion (the two terms are used interchangeably in the literature), defined as a head trauma resulting in brief loss of consciousness and/or alteration of mental state [1], is much harder to objectively detect. Concussion causes no gross pathology, such as hemorrhage, and no abnormalities on a conventional computed tomography scan of the brain [1], but rather rapid-onset neuronal dysfunction that usually resolves over a few days to weeks. Presumably, exposure to head injury has been a feature of human existence for millennia, and the human brain has evolved normal adaptive and recovery processes.

A chronic syndrome, likely related to repetitive concussions, has not received much attention until recently. However, in 1928, Martland published a case series describing symptoms such as slowed movement, tremor, confusion and speech problems in a group of former boxers, which he called ‘the punch drunk syndrome’ [2]. A few years later, Millspaugh called this syndrome dementia pugilistica [3]. In 1973, Corsellis and colleagues published a case series in which the brains of 15 retired boxers were studied and the neuropathology of what we now know as chronic traumatic encephalopathy (CTE) was first described [4]. The molecular pathology of this condition resembles aspects of Alzheimer's disease, but with some distinctions discussed below. Boxing was and still is the particular sport in which athletes voluntarily expose themselves to repetitive concussion. However, a number of other sports, for example, American football, ice hockey, rugby and martial arts other than boxing, have now developed in such a way that professional participation involves a considerably increased risk of repetitive concussions. More recently, case reports have shown pathologically confirmed CTE in contact sports athletes other than boxers and in former combat military personnel [5–7]. This has resulted in renewed interest in the potential for long-term neurodegenerative changes to occur after concussive and subconcussive repetitive head trauma [8]. In addition to case reports showing pathology in ex-sportsmen, there is epidemiologic evidence suggesting increased dementia risk after documented head injury [9], but apart from these types of descriptive studies there is a lack of systematic research on the problem.

Neuronal dysfunction in concussion is at least partly caused by direct damage to axons and other structures in the CNS. Approximately 15% of concussion patients suffer persisting cognitive dysfunction [10,11] and diffuse axonal injury (DAI) appears to be the most important underlying pathology in such cases [12]. The dysfunction may also relate to tau pathology that spreads around the brain in CTE [13,14]. However, what initiates this destructive and neurotoxic spreading cascade only in some individuals and not others is unknown.

It is important to remember that head injury and concussion are not synonyms (one head injury may not impact the brain at all, whereas another, depending on how the mechanical forces of the head blow are transferred to the brain, may impact the brain profoundly), which may be a problem in epidemiological studies. It is also clear that different sports and other risk activities have different types of contact and, therefore, probably different types of lesion. What are the damaging types of lesion and what types of damage are reversible and what are not reversible? How long does it take to recover from any particular type of damage? What are the outcomes of playing long-term professional sports today, when everything is faster, the players are heavier and the protective gear for the head appears less effective than protective gear for other parts of the body?

We now need systematic research on injury mechanisms in concussion and how they relate to CTE. Fluid biomarkers in TBI may relate directly to brain injury, released principally from neurons, glia or blood vessels or to response mechanisms such as microglial and astrocytic migration and activation, which may be protective or deleterious. Most individuals with concussion do not develop CTE. However, in some individuals there may be a transition from an injury/recovery pattern to a persistent, progressive process representing the initiation of a neurodegenerative process and a transition to the earliest phases of CTE. We hypothesize that this may relate to the extent, frequency and cumulative effect of brain injury together with variation in individual vulnerability to injury. It is possible that intrinsic (genetically determined) risk factors for Alzheimer's, Parkinson's or tauopathies such as progressive supranuclear palsy will also be risk factors for the development of CTE. Identification of individuals in the earliest phases of CTE is of crucial importance in terms of instituting appropriate advice on subsequent exposure, and in selecting individuals for future trials of disease-modifying therapies. Fluid biomarkers have the potential therefore to help to define: the severity of TBI; adaptive and recovery processes following TBI; and the transition between a normal injury/recovery pattern and a currently irreversible and inexorable CTE process. In this review, we start by discussing the different fluids that may be used as samples in which concussion biomarkers may be measured. We thereafter discuss technical aspects of the biomarker analysis. We then review the literature published so far on candidate biomarkers for concussion. Finally, we discuss the special case of CTE and emphasize the need for biomarker development for this disease entity.

## Marker matrices

### Cerebrospinal fluid

Cerebrospinal fluid (CSF) is a clear fluid that surrounds the brain, provides mechanical support and helps clear metabolites from the brain parenchyma together with direct transport across the blood–brain barrier, clearance via the glymphatic system and clearance via the recently discovered meningeal lymphatic vessels [15,16]. The total CSF volume is approximately 150 ml and the production and clearance rates are approximately 20 ml/h. Twenty to 30% of the CSF volume is derived directly from the brain; 70–80% is a choroid plexus-derived filtrate of plasma. CSF is sampled through a lumbar puncture, which is a harmless procedure with postlumbar puncture headache as the sole potential complication [17]. Standard operating procedures for CSF sampling and handling have been established and the procedure can be done in outpatients [18]. The main advantage of CSF as a matrix in which to measure markers of CNS injury is that it communicates freely with the brain interstitial fluid that bathes the neurons. Biochemical changes in the brain are thus reflected in the CSF, which may be regarded as an accessible, although by no means perfect, sample of the brain interstitial fluid. Further, CSF has low protease activity and most molecules do not change upon sampling provided the sample is not contaminated by blood. The main disadvantage is that lumbar puncture may be regarded as impractical to perform in emergency settings and in clinical studies. Another limitation is that it is presently unclear how clearance of brain metabolites into the CSF relates to clearance via the glymphatic and/or lymphatic system of the brain; potentially, biomarkers we thought would be well reflected in the CSF may escape detection if their main clearance pathway is through these newly described systems directly into the blood.

### Blood

The other major biofluid for the measurement of concussion markers is blood (serum or plasma). Blood is more accessible than CSF but most CNS-enriched markers are present in blood at very low concentrations that necessitate the employment of ultrasensitive techniques that can measure in the femtomolar range (most standard immunochemical techniques cannot reach this analytical sensitivity). The blood–brain barrier also poses a challenge in the analysis of CNS injury markers in blood. Normally, the blood–brain barrier restricts the release of CNS-enriched proteins and peptides into the bloodstream and, for some molecules, specific transport mechanisms exist. In concussion, there may be a transient opening of the blood–brain barrier, which could increase the blood concentrations of CNS-enriched molecules, also in the absence of direct injury to the structures they are thought to represent. It may thus be difficult to tell to what extent a peak in the blood concentration of such a biomarker reflects CNS injury or blood-brain barrier damage/dysfunction. Reliable blood biomarkers for blood–brain biomarker integrity relatable to CNS injury markers would be a major contribution to the field (the best established biomarker so far for blood–brain barrier function is the CSF/serum albumin ratio, which obviously necessitates access to both body fluids [19]). Further, most intracellular proteins released into the bloodstream undergo degradation and/or modification by proteases and other enzymes and for most of the biomarker candidates discussed below, the normal half-life is unknown. The dilution of CNS proteins into 4 l blood instead of 150 ml CSF may also contribute to the low concentrations of CNS-derived molecules in the blood.

### Saliva, urine & tears

It is possible that some CNS-derived proteins are eventually excreted into body fluids other than CSF and blood. The presence of the axonal protein tau in saliva has been demonstrated using mass spectrometry [20]. The same research group has also detected Parkinson-related α-synuclein and DJ-1 in this body fluid [21]. However, the relationship between salivary concentrations of these proteins and processes within the CNS is far from clear and no conclusive data on disease association have been reported so far. Similar lines of reasoning are relevant to tears and urine, although renal clearance of potential concussion biomarkers may be a more physiological pathway than salivary or lacrimal clearance.

## Measurement techniques

Most fluid markers of CNS injury are proteins or protein fragments that can be measured using immunochemical or MS-based techniques (or combinations thereof).

ELISA has become established as a standard method for the measurement of proteins in biofluids. The general principle is that a capture antibody directed against one epitope on the target analyte is immobilized on a surface, whereafter sample and labeled detector antibody (directed against another epitope on the same analyte) are added sequentially between blocking and washing steps to remove unspecific signal. Capture and detector antibodies are in molar excess so that most of the target analyte is captured in a sandwich between the antibody pair. Many ELISAs and ELISA-like techniques can reach lower limits of quantification of 10–100 pg/ml, but measuring even lower concentrations, as is needed for most brain-specific proteins in the blood, is a challenge. Auto-antibodies against the target analyte or heterophilic antibodies (e.g., endogenous anti-mouse IgG antibodies) that may react with the antibodies in the assay may block epitopes or bridge the capture and detector antibodies (replacing the analyte), giving falsely low or high signals. Interference from heterophilic antibodies may be blocked using polyclonal mouse IgG or commercially available blockers.

To allow for ultrasensitive measurement, two new techniques have entered the market: Erenna and Simoa [22]. The magnetic bead-based Erenna system can detect molecules at femtogram/ml concentrations using Single Molecule Counting (SMC) technology in which labeled detector antibodies are released from the captured immunocomplexes and counted one by one. Simoa is based on the isolation of individual immunocomplexes on magnetic beads using standard ELISA reagents. The main difference between Simoa and conventional immunoassays lies in the ability to trap single beads in femtoliter volume wells. This compartmentalization of the detection reaction allows for a digital readout of each individual bead to determine if it has bound the target analyte or not. In theory, this type of assay thus measures at the single molecule level, which may also be true when using the SMC approach of Erenna. Another upcoming technique for ultrasensitive biomarker quantification is proximity ligation assay, which builds on the principle that recognition of target proteins by two, three or more antibodies can bring in proximity DNA strands conjugated to the antibodies. The DNA strands can then participate in ligation reactions, giving rise to molecules that can undergo rolling circle amplification for highly sensitive detection [23]. The same potential interferences as for ELISA, applies to these types of measurement techniques as well, as they are all antibody based.

MS-based explorative proteomics has been applied to discover novel biomarkers in complex samples such as CSF and plasma for many years. More recently, however, antibody-independent selected or parallel reaction monitoring (SRM or PRM)-based MS techniques have been developed for the quantitative measurement of proteins and protein fragments in a manner that is stable enough to allow for use on large sample series and in clinical laboratory practice [24]. SRM-based MS is a method that can be expected to grow into a complementary or alternative technique to immunochemical assays in the analysis of protein markers in the near future [25].

## Fluid markers of acute mild traumatic brain injury

**Figure 1.**
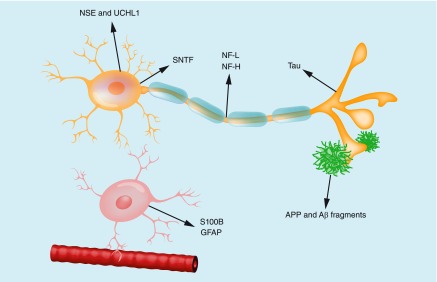
**A neuron, an astroglial cell, a blood vessel and diffuse Aβ deposits close to a synapse.** Candidate fluid biomarkers for concussion are indicated. NSE is a protein highly expressed in the neuronal soma, but also in red blood cells. UCHL1 is a de-ubiquitinating enzyme highly expressed in neurons, but also in gonads and lung tissue. α-SNTF is an axonal injury marker generated by the calpain family of calcium-activated proteases. SNTFs accumulate in the neuronal somaproximal part of injured axons following traumatic brain injury. NF-L and NF-H are intra-axonal structural proteins highly expressed in large-caliber axons. Tau is an intra-axonal structural protein highly expressed in thin, unmyelinated axons. S100B and GFAP are astroglial proteins. S100B is CNS-enriched but not specific, while GFAP appears to be highly CNS-specific. Aβ and other APP fragments may represent increased amyloidogenic APP-processing and diffuse plaque formation in response to axonal injury in traumatic brain injury. Aβ: Amyloid β; NF-H: Neurofilament heavy; NF-L: Neurofilament light; SNTF: Spectrin N-terminal fragment.

Candidate fluid markers of acute mild TBI are summarized in Figure 1.

### CSF markers

Axonal injury in concussion can be identified and monitored using CSF levels of the intra-axonal proteins neurofilament light (NF-L) and tau [26,27], measured by ELISA. NF-L is a structural protein that is highly expressed in large-caliber myelinated axons that extend subcortically into deeper brain layers. These are the primary targets when a blow to the head applies rotational forces to the brain inducing DAI and NF-L leakage from injured axons into the CSF. After a boxing bout, NF-L concentrations in CSF correlate with the number of received head blows [26,27]. Similar results have been obtained using an assay for phosphorylated neurofilament heavy protein [28]. A recent case report on a knocked-out amateur boxer showed that it took 8 months before his CSF NF-L concentration normalized [29]. This result resonates with neuropathological analyses showing that axonopathy can continue for years after TBI [30].

Tau is primarily expressed in thin unmyelinated axons and may respond more to cortical contusions than rotational brain injuries. However, CSF concentrations of tau change after both concussive and subconcussive head blows in a manner similar to NF-L but with a lower amplitude in the changes [27].

Studies in TBI models and on human brain tissue samples, as well as in brain interstitial fluid have demonstrated that APP accumulates in neurons and axons after brain trauma with axonal damage and that there is release of amyloid β (especially aggregation-prone Aβ42) into brain interstitial fluid with plaque formation around damaged axons [31]. In spite of this, there are no clear changes in CSF levels of secreted APP or Aβ fragments in concussion [26,27].

### Blood markers

In regard to blood biomarkers for concussion, recent data show promising results for tau, when measured on the ultrasensitive Simoa platform discussed above. A tau assay has been established on this platform and the first pilot studies have shown: a strong correlation between serum tau concentrations and neurological outcome in resuscitated cardiac arrest patients [32], increased tau concentrations in plasma from Olympic boxers [33], increased tau concentrations in plasma from patients with Alzheimer's disease [34], increased plasma tau concentrations in concussed ice hockey players 1 h after the injury; the degree of tau elevation correlated with the number of days it took until the players were free from symptoms [35]; and increased tau concentrations in plasma from military personnel who had been deployed within the previous 18 months and reported or had medical records on having had a TBI [36]. The dynamics of tau changes in blood and CSF following acute brain injury appears to be distinct; tau levels in CSF and plasma do not correlate [34] and tau elevations following acute brain injury stay much longer in CSF (weeks) than in blood (days) [32,37]. Possibly, tau is degraded when entering the bloodstream. A recent study by Al Nimer *et al*. used conventional ELISA for detection of NF-L in serum of TBI patients, showing increased serum concentrations in patients with the most severe neurotrauma [38]. The analytical sensitivity of the assay would, however, not allow for measuring serum NF-L in patients with mTBI or concussion and the correlation with CSF NF-L concentrations was very low, in contrast to a recently published ultrasensitive assay based on the Simoa platform [39], which is now being examined in relation to TBI.

Another promising blood marker of concussion is the 1176 residue N-terminal fragment of α-spectrin, termed SNTF, which is a protein that accumulates preferentially in damaged axons [40]. SNTF is normally undetectable in axons, but is generated following stretch injury by intra-axonal calcium overload and spectrin proteolysis mediated by the calpain family of calcium-activated proteases [41,42]. SNTF increases measurably in the blood after TBI, including CT-negative mTBI [43,44], and was recently found to be a predictive marker in sports-related concussion [45].

Other candidate blood biomarkers for brain injury in concussion include NSE, UCHL1, as well as the astroglia-enriched S100B and GFAP [46]. Clinically relevant changes in serum concentrations for S100B [47] and GFAP [48] have been reported to detect radiographically apparent intracranial injury and the former protein has been included as a biomarker that could reduce the number of unnecessary CT scans of the brain in new clinical guidelines for the management of head injury [47]. In contrast to tau and SNTF, none of these markers has a prognostic relationship with patient outcomes in concussion with negative brain computed tomography scan findings [46]. NSE, UCHL1 and S100B are also expressed in extracerebral tissues, which restricts their interpretability in multitrauma [46].

## CTE

CTE is a neurodegenerative disease associated with repetitive head trauma [49]. Although initially believed to affect only boxers, the at-risk population has expanded to encompass a much wider demographic, including American football players, ice hockey players, wrestlers and military veterans. This expansion has garnered considerable media attention and public concern for the potential neurodegenerative effects of head trauma. There are no established autopsy-verified clinical criteria for CTE and no neuroimaging or fluid markers of the disorder, although the gross morphological changes seen in advanced CTE may be visualized using standard neuroimaging techniques [50]. The molecular pathology of CTE is characterized by tau-positive neurofibrillary tangles and neuropil threads that may be present in all regions of the brain with or without Aβ pathology [49]. Neurofibrillary tangles in CTE form preferentially in the superficial cortical layers, rather than in deeper layers as is more common in AD, with focal accumulations at the depths of the sulci [51]. Another distinctive feature is perivascular tau deposition [51].

In regards to *in vivo* markers of the molecular pathology in CTE, preliminary findings from positron emission tomography (PET) scanning using PET ligands for brain tau in retired national football league players have recently been reported; five retired players were compared with five matched controls without a concussion history and displayed higher overall signals of tau deposition in their brains [52]. Amyloid PET imaging in traumatic brain injury has yielded ambiguous data, possibly due to the diffuse nature of trauma-related Aβ deposits that are less prone to bind amyloid ligands than neuritic plaques [53].

No data on fluid markers of tau and Aβ pathology have been presented so far but clinical biomarker studies with longitudinal follow-up of patients with chronic or progressive symptoms after TBI, for example, the Diagnosing and Evaluating Traumatic Encephalopathy using Clinical Tests (DETECT) study [54], are in progress and will tell us more about the pathogenesis, risk factors and clinical course of CTE, and how CTE can be diagnosed and monitored with the help of biomarkers.

## Could markers of mild TBI/concussion help prevent CTE?

The primary goal of biomarker research in TBI is not to develop techniques to identify moderate to severe brain injury, as such already exist, but rather to identify molecular changes in mTBI/concussion to indicate if the brain was injured by a head injury and to monitor the recovery process. Most current research suggests that the risk of long-term symptoms following concussion is highest in individuals who have received repetitive concussions before the brain has recovered properly [49]. Identifying incomplete recovery following concussion using an objective test and prolong the rehabilitation phase in such cases could potentially help to reduce the incidence of CTE.

## Conclusion & future perspective

Several promising CSF and blood markers of concussion exist but the field is not yet mature enough to rank them according to diagnostic accuracy. Their potential predictive value in regard to time to complete recovery and risk of incomplete recovery/CTE needs to be established. Further, we need more knowledge on release and clearance mechanisms of the candidate biomarkers and how their blood concentrations relate to blood–brain barrier dysfunction. Another outstanding research question is if additional information could be gained by combining different markers with each other, for example, by quantifying the extent of both the axonal injury and the inflammatory response using different biomarker combinations. All this should be relevant not only for sports-related concussions but also for the many concussions that are not sports related. Information gained using biomarkers for TBI could potentially be used to identify at-risk cases most appropriate for enrollment in clinical research studies and therapeutic trials.

**Executive summary**

- Concussion is difficult to diagnose clinically.
- Objective biomarker tests for brain injury in suspected concussion would be an important diagnostic tool.
- Such biomarker tests could also help to tell when the injurious process has resolved.
- Several cerebrospinal tests for brain injury in concussion are at hand.
- Ultrasensitive assays have made it possible to measure CNS-specific proteins in the blood.
